# A New Enterprise

**Published:** 1876-10

**Authors:** 


### *A NEW ENTERPRISE.*

GEORGE A. DEWEY, M.D., has established an agency in this city for the purchase of drugs and medicines, surgical instruments, Patent Medicines, etc., to order. An extensive practice of more than twenty years has demonstrated the importance of procuring pure medicines in cases of sickness. A large proportion of those sold by second-class druggists are adulterated to such an extent as to be worthless. It is, therefore, a matter of interest to every one to have such medicines as they may require selected by an educated and competent physician of experience. Dr. Dewey proposes simply to purchase medicines, surgical appliances, and all other things necessary for the sick, and not to continue the practice of medicine. He will, however, when the choice is left to him, select such remedies as he deems the best, when the nature of the disease is explained to him. He is, to a great extent, a believer in "specifics," that is, that nearly all diseases can be CURED by the use of the proper remedies.

All medicines or other articles will be furnished at the regular retail price in New York. No charge will be made for advice when the nature of the disease is stated, and the selection of the appropriate remedies is left to him; but with the money for the medicine, stamps or money must be sent to pay the postage—one cent for every ounce—on such articles as can be sent by mail. The charges on articles sent by express will be collected of the persons receiving them.

### DIRECTIONS.

When medicine, etc., are desired, write your order, naming the medicine you wish; or if you are sick and desire the best remedy, carefully state just what the trouble is, so far as you know, and give sex, age, and general condition of health. Inclose a postage stamp or postal card for reply. You will be promptly advised of the best remedy and the cost of it. All communications confidential. Address, Geo. A. Dewey, M.D., 137 Eighth Street, New York.

